# Investigating the Effects of Virtual Reality-Based Training on Balance Ability and Balance Confidence in Older Individuals

**DOI:** 10.3390/app14114581

**Published:** 2024-05-27

**Authors:** Oshin Wilson, Nicole Arnold, Lara A. Thompson

**Affiliations:** Center for Biomechanical and Rehabilitation Engineering, Biomedical Engineering Program, School of Engineering and Applied Sciences, University of the District of Columbia, 4200 Connecticut Ave. NW, Washington, DC 20008, USA;

**Keywords:** aging, elderly, virtual reality, balance training, balance confidence, falls

## Abstract

Each year, over 25% of adults aged sixty-five years old or older suffer a fall, and three million are treated for fall-related injuries due to lack of balance. Here, we aimed to investigate how virtual reality (VR)-based training affects balance performance and confidence in older adults. To accomplish this goal, we studied 21 healthy, older individuals between 60 and 85 years old, both pre- and post-training (6 weeks of training, twice per week (or 12 sessions)). The VR group donned an Oculus VR headset and consisted of nine participants (aged 75.9 ± 3.7 years old), while the control group (aged 75.1 ± 6.7 years old) performed training without a headset and consisted of eight participants that completed our study. To assess balance ability, we utilized the Balance Error Scoring System (BESS) and the Timed Up and Go (TUG) test. To assess balance confidence, we implemented the Activities-Specific Balance Confidence (ABC) Scale and, to assess fear of falling, the Tinetti Falls Efficacy Scale (FES). Further, we assessed depression (via the Geriatric Depression Scale (GDS)) and cognitive ability (via the Mini-Mental State Examination (MMSE)). The post-training results showed improvements in balance ability for both the VR and control groups, as well as changes in the relationship between balance confidence and balance ability for the VR group only. Further, improvements in cognitive ability were seen in the control group. This study is an indication that older individuals’ balance ability may benefit from several weeks of targeted training.

## 1. Introduction

Rapid increases in the number of aging individuals worldwide make it critical to identify interventions and strategies toward maintaining and improving older adults’ balance, both for general health and to avoid fall-related injury and death. While traditional methods of balance training have demonstrated efficacy, there are still issues with participant engagement and realistic scenario simulations. In light of this, virtual reality (VR), with its capacity for immersive, simulated environments, appears to be a potential means of advancing balance training among older adults. VR provides a dynamic platform that can create engaging environments for balance exercises; its ability to simulate real-life scenarios allows older adults to practice balance techniques in varied and challenging settings, enhancing adaptability and transferability to daily activities.

The fear of falling is a major concern that increases as individuals age. Fear of falling (FoF) is defined as an enhanced concern about falling that creates anxiety toward and avoidance of daily activities [1]. This fear often arises, but not always, due to previous falls or near-falls, physical frailty, or a perception of diminished balance or mobility. FoF can lead to restricted activity levels, social isolation, and a decrease in overall quality of life. Balance confidence and FoF are closely related concepts that play significant roles in the well-being and functional independence of individuals, particularly among older adults. Balance confidence is an individual’s confidence and perceived ability to maintain their balance while performing various activities [2]. Some of these activities may include walking, climbing stairs, or reaching for objects. It encompasses a sense of assurance and self-efficacy in one’s balance abilities.

To investigate the effects of VR training on balance ability and FoF among older adults, numerous studies have explored its potential benefits [3,4]. However, existing research often falls short of addressing certain gaps within this domain. While some studies have demonstrated the efficacy of VR in enhancing balance among older adults, many have failed to comprehensively assess factors such as stability, balance confidence, cognitive abilities, and postural control using objective measures such as the Balance Error Scoring System (BESS) [5], the Activities-Specific Balance Confidence (ABC) Scale [6], and the Mini-Mental State Examination (MMSE) [7], among other significant assessments.

Previous VR balance studies have not investigated balance confidence or depression, though some have explored cognitive ability [4,8]. These studies have consistently demonstrated significant enhancements in postural control, balance, gait, and functional mobility among older adults following VR-based interventions. However, none of these studies have investigated the impact of VR interventions on variables such as balance confidence in older adults. The use of VR training to help older adults with their balance issues has generated our research question on how VR training affects balance ability, balance confidence, and the relationship between the two. Our investigation aims to fill gaps in previous studies by examining the effects of several weeks of VR-based training on both balance confidence and ability in older adults. Specifically, we hypothesize that balance confidence will increase and FoF will decrease post-training. Additionally, we expect improvements in balance ability, measured through validated assessments. Further, the relationship between balance ability and confidence will change for those who receive VR-based training. This research provides valuable insights for healthcare practitioners, researchers, and policymakers seeking evidence-based strategies to mitigate falls and enhance the well-being of older adults. By incorporating a control group, something overlooked in previous studies, for comparative analysis and utilizing validated assessments, we aim to comprehensively evaluate the impact of VR training on both balance confidence and FoF.

## 2. Materials and Methods

This experimental clinical trial study was conducted at the University of the District of Columbia (UDC), within the Center for Biomechanical and Rehabilitation Engineering (CBRE). The project was approved by the Institutional Review Board (IRB) (2073871–01). The UDC Center for Advancement of Learning (CAL) provided Oculus headsets that were used during the VR training.

### 2.1. Study Overview

**Figure 1.**
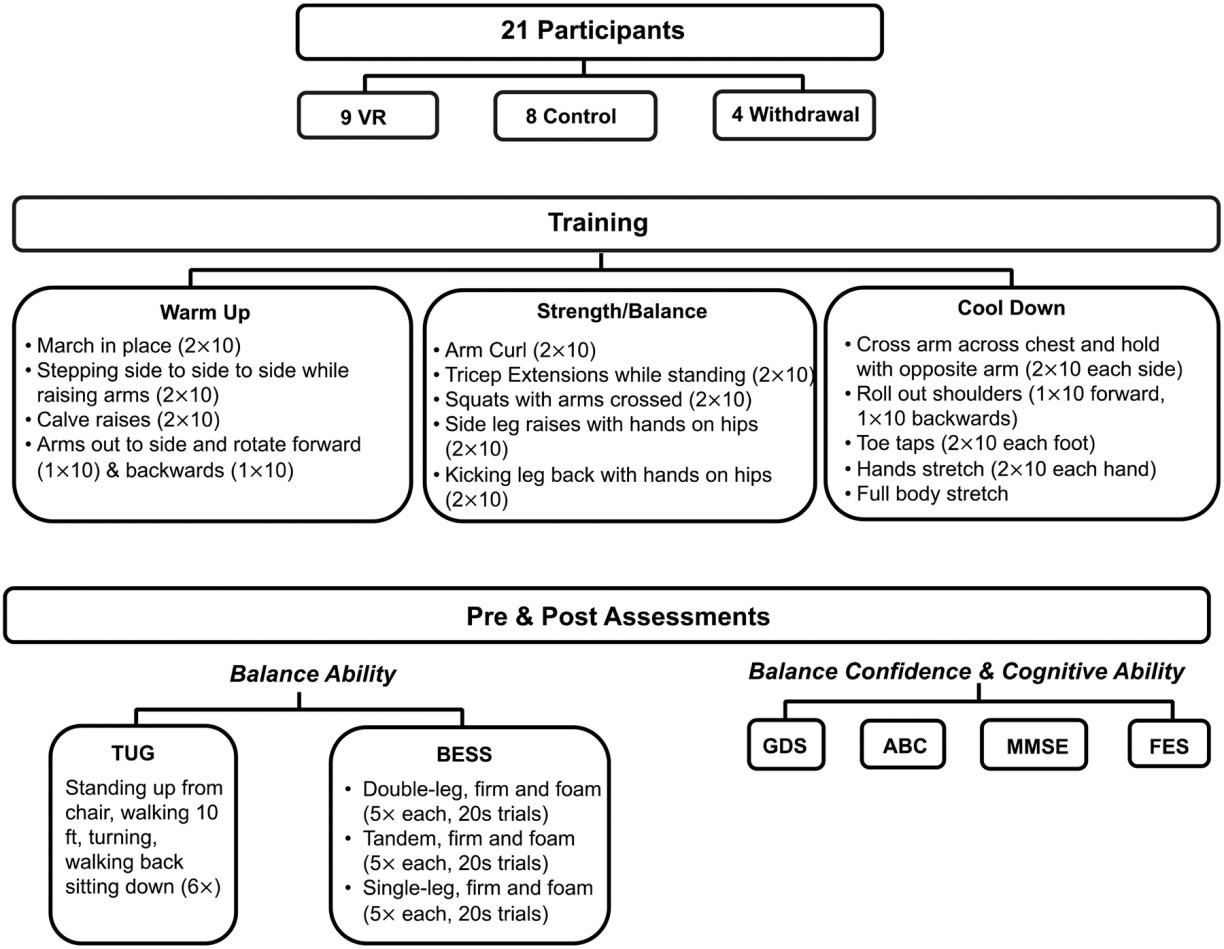
Overview of research study.

A study overview is shown in Figure 1. Participants, training, and pre- and post-training assessments are discussed in the sections below.

#### 2.1.1. Participants

Participant demographics are shown in Table 1. Participants provided their informed consent prior to taking part in this research study. Participants were recruited via a multifaceted approach that included flyers posted around the campus and spreading the word through informal channels such as word of mouth. The participants were randomly selected and placed into the different groups; however, some indicated their preference. There were two groups of participants: the experimental group (persons performing the training with the VR headset, or VR group) and the control group (persons performing the training without the headset). There were 21 participants in this study; however, 4 withdrew because of work, travel, and other engagements, leaving 9 participants in the VR group (75.9 ± 3.7 years old) and 8 participants in the control group (75.1 ± 6.7 years old). With 10 being the most fit and 1 being the least fit, for the VR group, their self-reported fitness level was 7.0 ± 1.5, and for the control group, their self-reported fitness level was 7.4 ± 1.7.

Inclusion criteria for this study encompassed older adults, both male and female, aged 60 to 85 years old, who defined themselves as healthy, possessed adequate cognitive function to comprehend instructions and engage in study activities, had sufficient vision and hearing, and demonstrated the ability to ambulate without a cane or wheelchair, without a significant physical disability. Exclusion criteria involved individuals experiencing excessive pain in their legs or feet, those with serious uncontrolled medical problems, and those unable to ambulate without assistance.

#### 2.1.2. Training

During our training program (two 30′ sessions per week for six weeks, or twelve training sessions), exercises targeted specific aspects of balance, such as static and dynamic balance, sensory integration, and motor coordination (summarized in Figure 1, middle) for both the control and VR groups. The training exercise program was formulated using a video created by the National Institute on Aging as a guide to workouts geared towards balance exercises for older adults [9].

**Figure 2.**
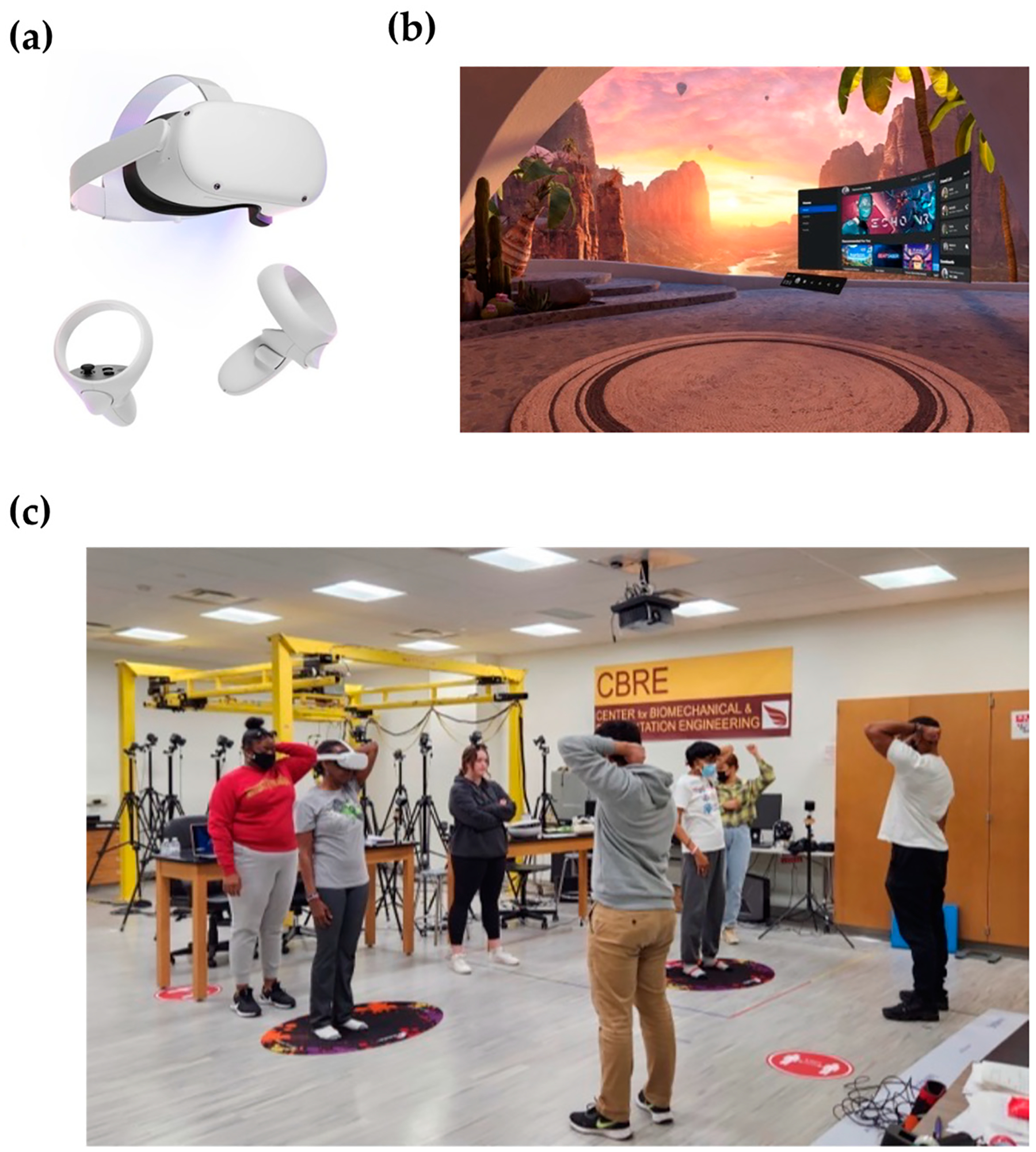
(**a**) Oculus VR headset; (**b**) VR headset environment; (**c**) example of VR and control group training in UDC CBRE.

The Oculus VR system (Figure 2a) was used during our training of the older participants within the VR group. The VR system would provide a safe and controlled environment (scene shown in Figure 2b) for the participants to practice different strength and balance exercises. An example training session is shown in Figure 2c.

#### 2.1.3. Pre- and Post-Training Assessments

**Figure 3.**
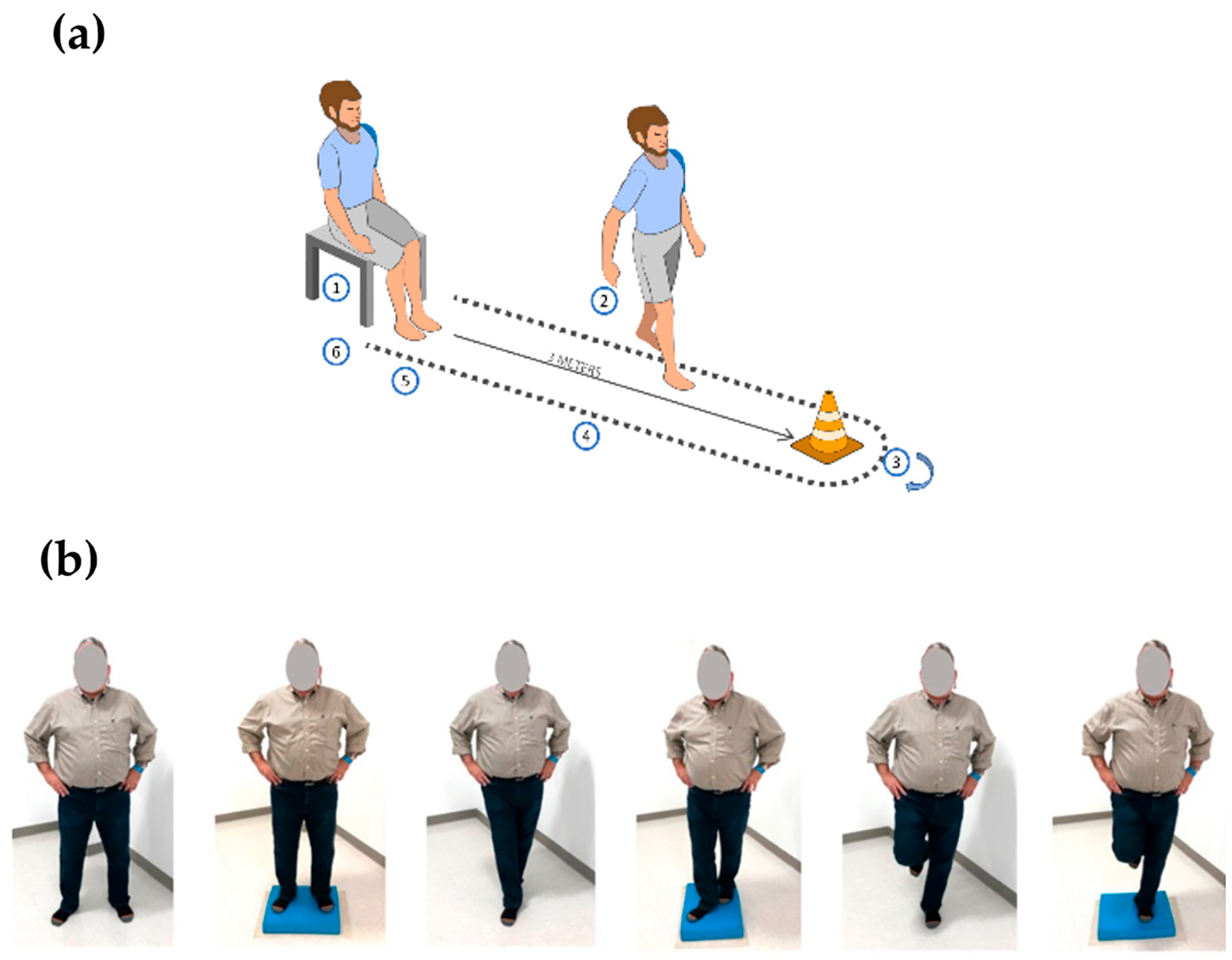
(**a**) TUG [12] and (**b**) BESS assessments [10].

Assessments were conducted pre-training (or at 0 weeks) and post-training after completing 12 sessions (or at 6 weeks). Participants were blinded to their pre-training assessment results when attempting the post-training assessment. Our study evaluated balance using a multifaceted assessment approach. To assess mobility and standing balance, the Timed Up and Go (TUG) test and the Balance Error Scoring System (BESS), respectively, were examined. The TUG was used to evaluate pre- and post-VR training functional capacity, mobility, and dynamic balance in our study. The TUG test involved timing how long it took the participant to: (1) get up from a chair, (2) walk 10 ft, (3) turn around, (4) go back, (5) stop and (6) sit down (Figure 3a). The threshold value for fall risk is ≥13.5 s [10]; a shorter time to finish the TUG test suggests that the VR interventions have improved mobility, balance, and overall functional capacity.

To assess standing balance, measures were taken utilizing the BESS. The BESS is a widely employed clinical test for evaluating static balance and postural stability, encompassing double-leg, single-leg, and tandem stances on both firm and foam surfaces to challenge one’s balance during several (5) 20 s trials per stance [11]. This system is commonly used in, for example, concussion management, rehabilitation, and sports medicine to assess changes in balance and intervention effectiveness. The test conditions are shown in Figure 3b. Participants receive scores based on specific error criteria, such as opening eyes when instructed to be closed or stepping out of the designated stance. The scoring system quantifies errors made during each stance, with a lower score indicating, potentially, better postural stability. Errors are characterized by opening the eyes, removing hands from the iliac crest, taking a step to regain balance, stumbling or falling out of position, lifting the forefoot or heel, abducting or flexing the hip by over 30 degrees, or failing to return to the test position within 5 s [11].

To measure psychological and emotional aspects of balance and falling, we used the Activities-Specific Balance Confidence (ABC) Scale [13], the Tinetti Falls Efficacy Scale (FES) [6], and the Geriatric Depression Scale (GDS) [14]. The ABC scale is a self-reporting assessment designed to evaluate an individual’s confidence (0% meaning no confidence and 100% meaning completely confident) in their ability to carry out different tasks (both at home and outside) without falling or losing their balance. The questionnaire consisted of several questions that were geared towards how confident one was in doing activities in and out of one’s home. Some of the questions included the following: “How confident are you that you will not lose your balance or become unsteady when you: walk up or down the stairs? Reach for a small can off a shelf at eye level? Sweep the floor? Walk across the parking lot to the mall?”. The FoF was assessed by the FES (a 10-item questionnaire), where on a scale from 1 to 10, with 1 being very confident and 10 being not confident at all, the participants are asked about their fear of falling during in-home activities. Examples of scenarios are taking a bath or shower, reaching into cabinets or closets, and walking around the house. A total score of greater than 70 indicates that the person has a fear of falling [6]. The Geriatric Depression Scale (GDS) is a self-reporting measure of depression in older adults. Users respond in a “Yes/No” format [14]. The GDS, which consisted of 10 questions, included questions such as the following: “Are you basically satisfied with your life? Have you dropped many of your activities and interests?”. A score of 5 or more suggests depression. The ABC scale is a self-reporting assessment designed to evaluate an individual’s confidence in their ability to carry out different daily living tasks (both in and out of the home) without falling or losing their balance [13]. It is a comprehensive 16-item self-reporting measure designed to gauge individuals’ confidence in their ability to perform various activities without losing balance or feeling unsteady. Participants rate their confidence levels on a scale ranging from 0 to 100, where a score of 0 indicates no confidence, while a score of 100 signifies complete confidence. In addition, the Mini-Mental State Examination (MMSE) was used as a cognitive screening tool [7]. This is an 11-question measure that tests five areas of cognitive function: orientation, registration, attention and calculation, recall, and language. The MMSE survey included questions that involved tasks such as recalling a series of words, performing simple arithmetic calculations, naming objects, following verbal commands, and copying a simple figure. The total possible score is typically 30 points, with higher scores indicating better cognitive function and a lower score, often below 24 points, indicating cognitive impairment.

### 2.2. Analysis

To analyze the data, both pre- and post-averages and standard deviations across each group of participants were computed for BESS, ABC, FES, GDS, and MMSE. The statistical analysis was conducted using the RStudio Software (RStudio Version 2023.12.1, Boston, MA, USA). Student’s *t*-tests were used for normally distributed data to determine whether the means of the two groups were significantly or insignificantly different from one another. The assumption for the test was that both groups were sampled from normal distributions with equal variances. For non-normally distributed data, the Wilcoxon test was used, which is a non-parametric alternative to the *t*-test for comparing two means. These statistical analyses provided insights into the significance of changes pre- and post-training for both the virtual reality and control groups.

## 3. Results

**Figure 4.**
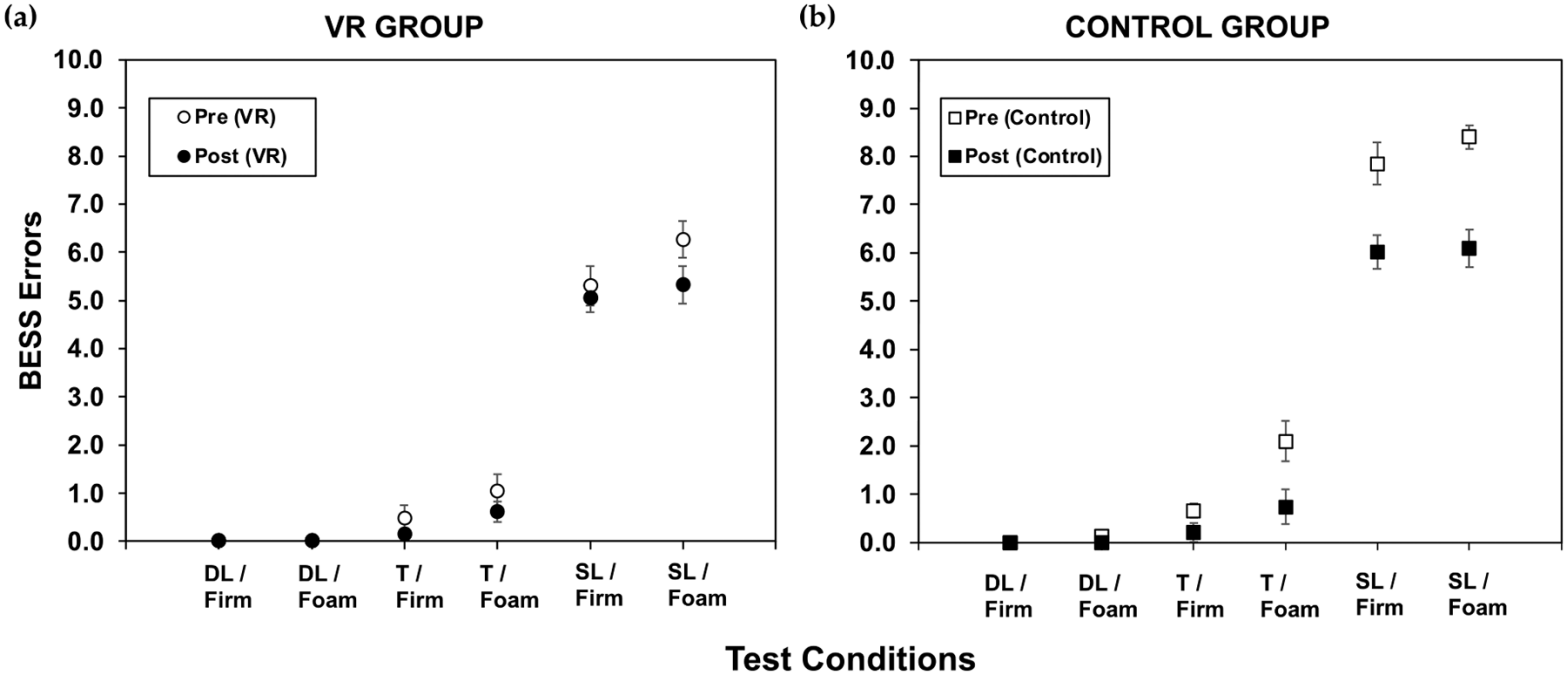
Total BESS errors for the (**a**) VR group and (**b**) control group pre-training (open icons) and post-training (filled icons) for double-leg/firm (DL/firm), double-leg/foam (DL/foam), tandem/firm (T/firm), tandem/foam (T/foam), single-leg/firm (SL/firm), and single-leg/foam (SL/foam) stance conditions; pooled data with standard error bars shown.

The results for the BESS errors across stance conditions are shown in Figure 4.

Notable changes were observed in the control group’s balance performance, particularly evident in the reduction in errors across the various stances and surfaces. For SL/foam, there was a decrease in errors with a significant change (*p* = 9.20 × 10^−7^), and also for T/foam, with a significant change of *p* = 0.003. SL/firm also showed a decrease in errors, with a significant change of *p* = 0.0001.

The VR group only had a significant decrease in BESS errors for SL/foam, with a significant value of *p* = 0.001. For the changes observed in both groups for the different stances previously mentioned, a decrease in the BESS errors indicates an improvement in balance and stability.

**Figure 5.**
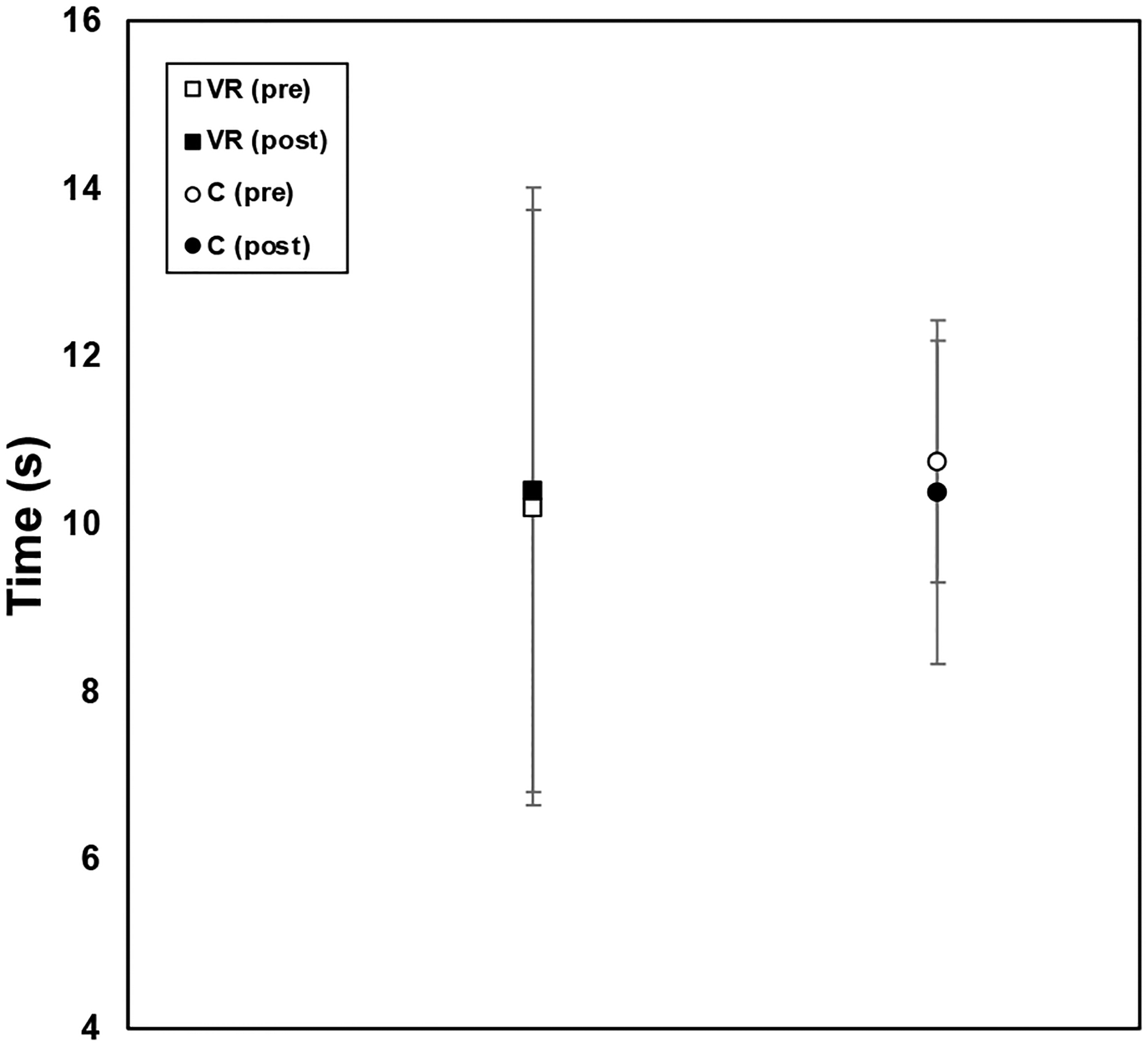
Timed Up and Go (TUG) results for VR group and control group pooled data, pre-training (open icons) and post-training (filled icons), with standard error bars shown.

According to the TUG assessment results (shown in Figure 5), for both the VR and control groups, there were no significant changes for the different groups of participants preand post-training. Prior expectations might have leaned towards the VR group showing more significant improvements given the immersive and engaging nature of VR training; however, based on the data, that was not observed.

**Figure 6.**
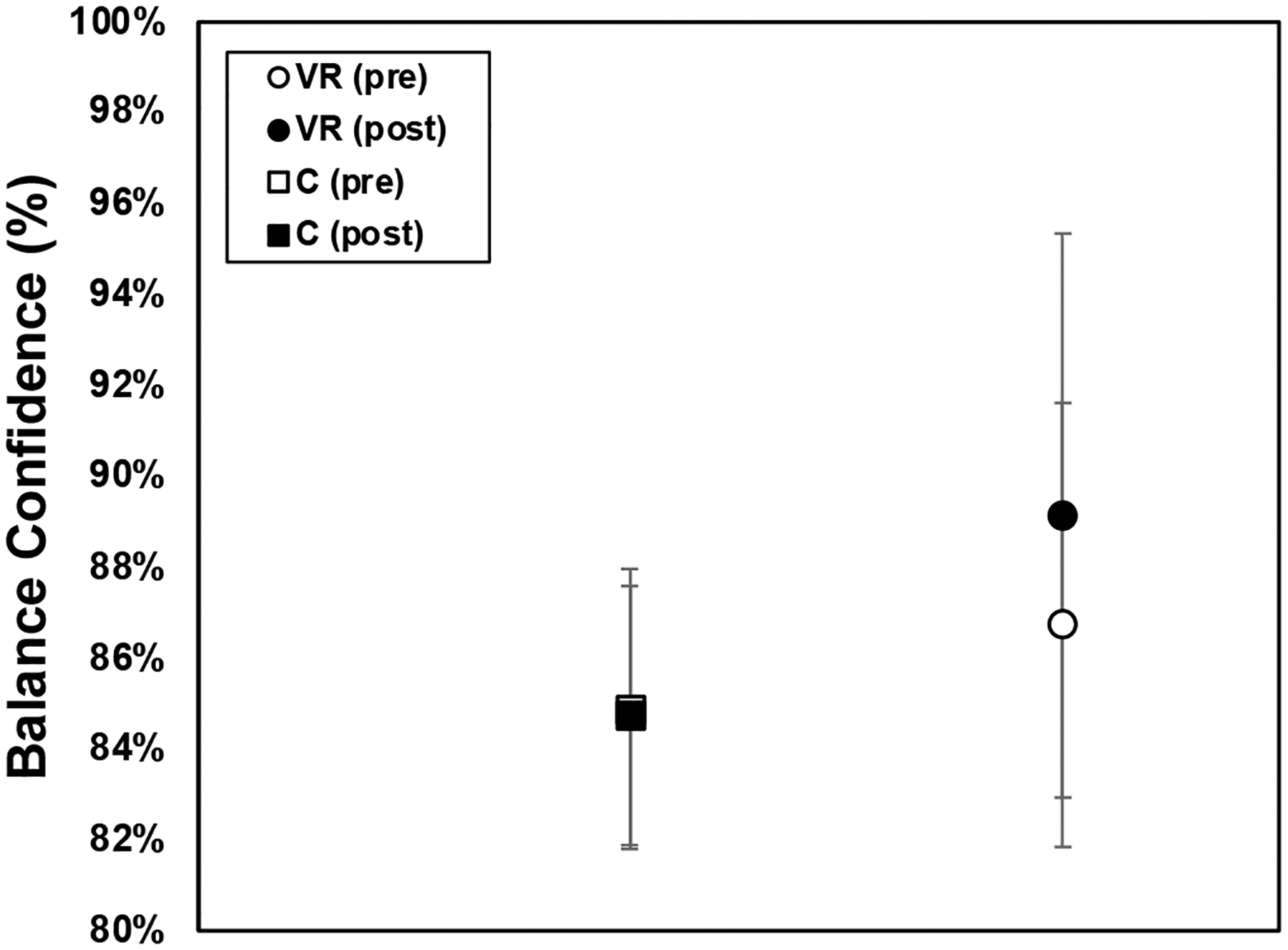
Activities-Specific Balance Confidence (ABC) results for VR group and control group pooled data, pre-training (open icons) and post-training (filled icons), with standard error bars shown.

**Figure 7.**
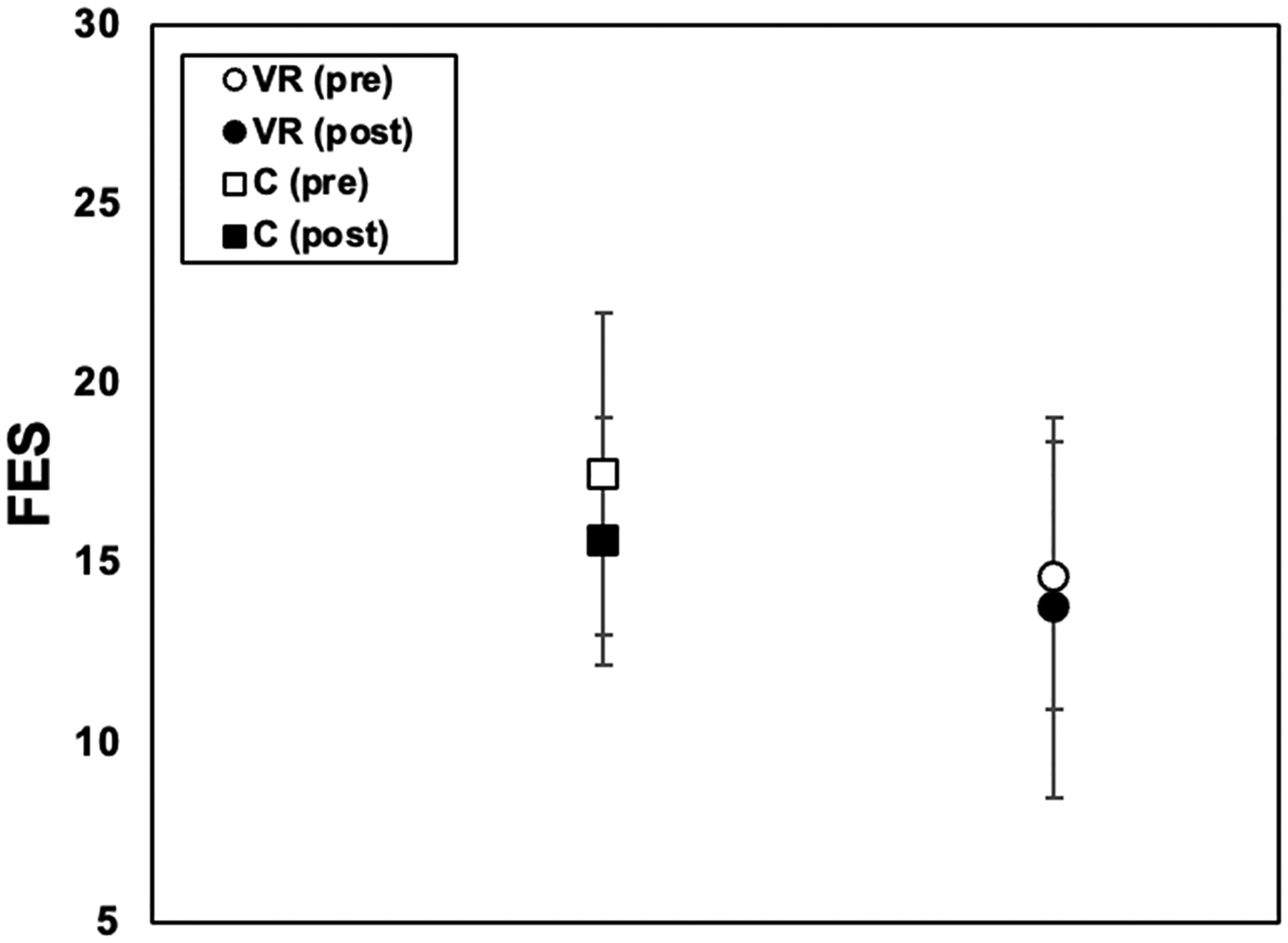
Tinetti Falls Efficacy Scale (FES) results for VR group and control group data, pre-training (open icons) and post-training (filled icons), with standard error bars shown.

**Figure 8.**
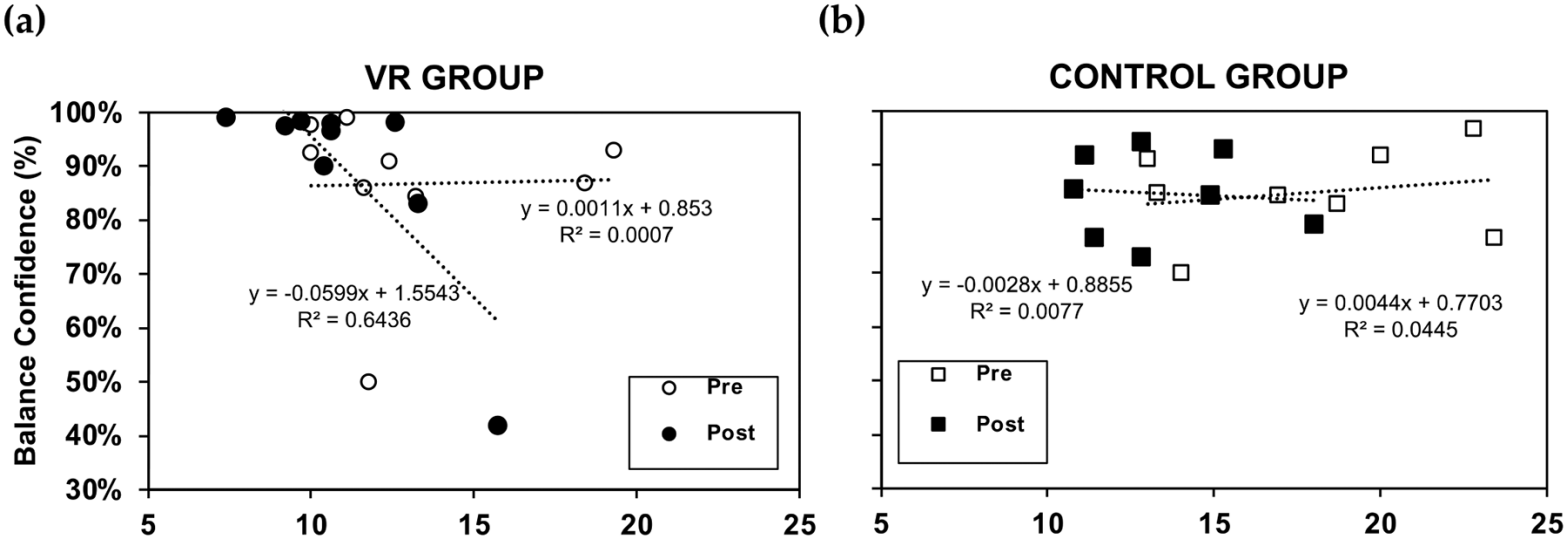
Activities-Specific Balance Confidence (ABC) results as a function of the Balance Error Scoring System (BESS) for (**a**) VR group and (**b**) control group, pre-training (open icons) and post-training (filled icons)

Below are the results for the ABC (Figure 6), FES (Figure 7), and ABC as a function of the BESS (Figure 8).

The results from the ABC Scale assessments reflected that the perceived balance confidence of the participants in both the VR and control groups before and after the training intervention did not change significantly. In the VR group, the average balance confidence was 86.73% ± 4.887 and 89.15% ± 6.201, while in the control group, it was 84.88% ± 3.075 and 84.76% ± 2.849 pre- and post-training, respectively.

The FES scores reflected the participants’ perceived ability to avoid falling during daily activities. For the control group, the mean pre-assessment FES score was 17.5 ± 4.4801, while the mean post-assessment was 15.63 ± 3.459. For the VR group, the mean pre-assessment was 14.67 ± 3.697, while the post-assessment was 13.78 ± 5.302. The changes seen were insignificant for both groups.

Figure 8 shows the ABC results as a function of the BESS. For the VR group (Figure 8a), prior to training, there was no correlation between balance confidence (i.e., ABC results) and balance ability (i.e., BESS results). However, post-VR training, decreases in ABC (i.e., worsening of balance confidence) with increases in BESS errors (i.e., worsening of balance ability) were observed; this indicates a negative correlation between the balance confidence and BESS scores. Conversely, increases in ABC (i.e., enhanced balance confidence) were associated with decreases in BESS errors (i.e., enhanced balance ability). In simpler terms, this means that when individuals have lower confidence in their balance abilities, they tend to make more errors, according to the BESS assessment. For the VR group, this implies that there is a relationship between one’s perceived confidence in their balance and their actual performance on balance-related tasks, with lower confidence associated with poorer balance control or stability, as indicated by higher BESS scores. For the control group (Figure 8b), there was no correlation between balance confidence and balance ability. This was true both pre- and post-training.

**Figure 9.**
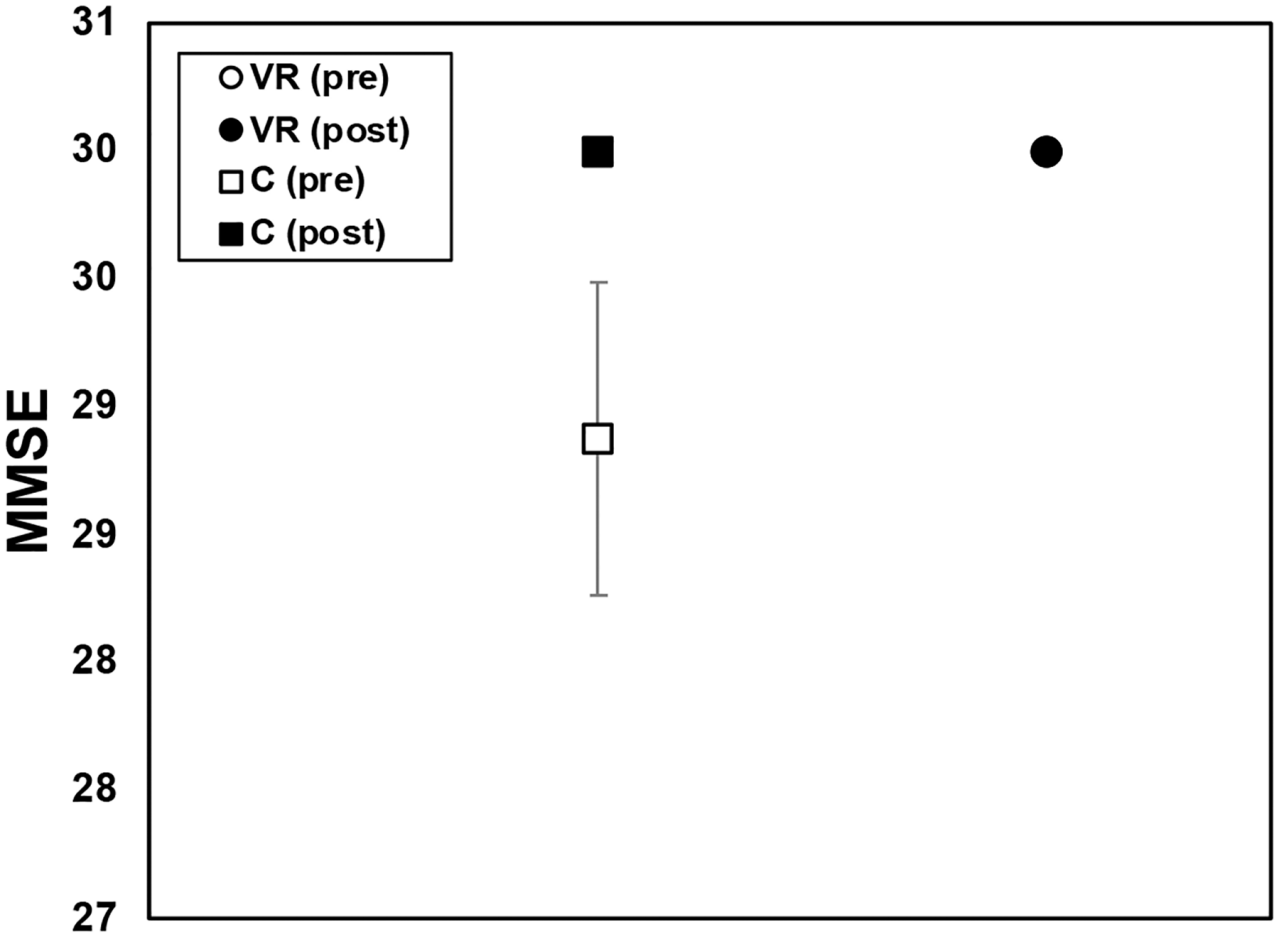
Mini-Mental State Examination (MMSE) results for VR group (circle) and control group (square), pre-training (open icons) and post-training (filled icons); pooled data with standard error bars shown.

Figure 9 shows the results from the MMSE for both the control and VR groups; both groups had high cognitive functioning among participants. The control group exhibited high MMSE scores both before and after the intervention, with an average pre-assessment score of 28.99 ± 0.612 and a perfect average post-assessment score of 30 ± 0. Remarkably, the standard deviation and standard error of the mean for the post-assessment scores were zero, indicating no variability in scores among participants after the intervention. This suggests that the control group maintained stable and high cognitive functioning throughout the study period, a result supported by a significance test (*p* = 0.032). This implies a robust confirmation of the control group’s cognitive stability and highlights the effectiveness of the intervention in maintaining cognitive health. The VR group exhibited excellent cognitive performance on the MMSE, with all participants scoring a perfect 30 ± 0 both before and after the intervention. The absence of variability in the scores, as indicated by the zero standard deviation and standard error of the mean, further implies the consistent cognitive functioning observed among the VR participants throughout our study. The high and consistent scores across assessments indicate that participants in both groups maintained optimal cognitive health and functioning throughout the study period.

The GDS scores shed light on the participants’ depressive symptoms and emotional well-being throughout our study. There were no significant changes observed for either group.

## 4. Discussion

Here, we aimed to investigate the effects of a VR-based training program on balance ability and balance confidence in healthy older adults pre- and post-training. The results of our study demonstrate that the VR training had an impact on balance performance in older adults; however, balance confidence and cognitive ability were not affected significantly. The control group also showed improvements in balance performance and cognitive ability, but not in balance confidence. For both groups, geriatric depression and falls efficacy did not change post-training.

The improvement observed in the BESS scores suggests that the VR training intervention was effective in helping participants enhance their balance skills during the training. A similar improvement is evident when previous studies in the literature are considered [2]. However, traditional training without a headset was also effective in improving balance. The BESS results revealed intriguing insights into the effectiveness of different training interventions in improving balance among participants. The decrease in BESS scores signifies an increase in balance stability. In terms of balance training, these findings not only have implications for individuals seeking to improve their balance but also for clinicians and researchers involved in designing and implementing balance training interventions. The results from the T/foam, S/foam, and S/firm stances in our study indicate that the VR group exhibited better balance performance compared to the control group prior to the training. However, following the six-week training period, the control group showed more pronounced enhancements compared to the VR group. This is evident given the significant values produced for T/foam, SL/firm, and SL/foam after testing, as seen in the results above for the control group.

In contrast, while the VR group also demonstrated changes in their balance performance post-training, the improvements were notable. Despite the control group’s improvement, the results still underscore the potential of VR training as a valuable tool for improving balance performance in older adults. The controls had slightly poorer performance on average prior to the training compared to the VR group; this could be why bigger changes were observed post-training. With the reduction in average errors in the S/foam stance, there is evidence of the potential of VR training in aiding older adults with their balance abilities. The immersive and engaging nature of VR environments, coupled with multisensory stimulation, likely contributed to the improvement observed in the VR group. Furthermore, VR training may offer additional benefits beyond traditional exercise methods, such as enhanced motivation and enjoyment, which could promote adherence to exercise regimens among older adults.

In light of previous research, the findings of our study align with the growing body of evidence supporting the efficacy of VR-based interventions in improving balance and reducing fall risk in older adults (e.g., [15]). Similarly, the significant reduction in errors observed in both the VR and control groups in the current study suggests the potential of exercise interventions, regardless of modality, in enhancing balance performance among older adults. The significant testing results for post- vs. pre-training assessments further support the effectiveness of both VR and traditional exercise methods in improving balance performance.

Given the immersive and engaging nature of VR training, our expectations leaned towards the VR group showing significant improvements (decreases) in the TUG; however, based on the data, that was not observed. There were no significant changes observed in the TUG results for both groups. It may be inferred that gait speed was also not improved by the training. In contrast to our study and training protocol, in other studies that employed the TUG assessment, significant improvements were observed [16]. The nature of our training was based on in-place balance exercises; thus, in future investigations, we could explore if more dynamic tasks would impact TUG and gait speed.

There was a lack of significance in the results for both groups in the ABC assessment after testing. A longer timeframe might have afforded participants more opportunity to adapt and enhance their confidence levels. Furthermore, considering the participants’ FoF prior to their involvement in this study, it is possible that some of the activity questions in the ABC assessment were outside their comfort zone due to their tendency to avoid such activities in their daily lives. For example, during the ABC assessments, it became apparent that when queried about their confidence levels in scenarios like walking outside on icy sidewalks without losing balance or becoming unsteady, most participants across both groups scored below 90%. This particular question appeared to pose significant challenges for all participants, indicating a common area of concern or struggle regarding balance confidence among the study cohort. It is worth noting that while our study’s findings are insightful, there is a scarcity of comparable research specifically focusing on balance confidence in older adults undergoing VR balance training, limiting direct comparisons with the existing literature.

While one study demonstrated significant changes in fear of falling [17], our study did not yield similar findings, suggesting potential variations in intervention modalities or participant characteristics across studies. The FES scores provided valuable insights into the participants’ perceived confidence in their ability to avoid falling during daily activities. The results for both groups in our study were insignificant after testing. This could be because of several factors. The variability in the participants’ responses to the intervention, including the differences in physical fitness or cognitive abilities, may have influenced the absence of significant findings. Individuals with diverse baseline characteristics might have responded differently to the intervention, impacting the overall effectiveness observed in this study. Moreover, the responses to questions on the FES are subjective, which could have introduced variability in the participants’ ratings. This subjectivity may have made it challenging to detect significant changes in FES scores, as perceptions of falls efficacy can vary widely among individuals. Addressing these factors is essential for interpreting this study’s outcomes accurately and understanding the potential nuances underlying the lack of significance in the FES scores.

The results for both groups were unchanged in the GDS assessment. A possible explanation for the lack of significant changes in the GDS assessment for both groups could be related to the nature of the training. The relatively short duration of the intervention (six weeks) might have been inadequate to induce noticeable changes in depressive symptoms, which often necessitate longer-term interventions or comprehensive mental health support.

It is noteworthy and reassuring that the MMSE scores either maintained or increased across both the control and VR groups throughout the study period. In concordance with our study, where cognitive function improvements were measured using the MMSE, a separate study employing the Montreal Cognitive Assessment (MoCA) also reported significant changes in cognitive function. However, while our study revealed enhancements in the control group, contrasting findings from the MoCA study showed improvements primarily in the intervention group [18]. The MoCA is a cognitive assessment tool comprising 30 dichotomous items, where each correct answer is assigned a score of 1 point. Total scores on the MoCA range from 0 to 30, with higher scores indicative of better cognitive function. The improvement or stability in the MMSE scores in both groups of our study suggests that the training had an effect on the participants’ cognitive functioning. This observation is particularly encouraging, as it indicates that the interventions did not exacerbate cognitive decline or lead to any adverse cognitive outcomes among the participants. Instead, the maintenance or enhancement of cognitive scores underscores the potential safety and positive impact of both the control and VR interventions on cognitive health in older adults.

### Limitations

VR studies with small sample sizes can offer valuable insights despite their limited participant pool [19]. While we acknowledge the potential limitations of an initially small sample size, small-scale VR studies can still provide significant findings, notably when they employ rigorous methodologies with thoroughly analyzed data. However, the size of the sample might have impacted the statistical power of the analyses, potentially limiting the ability to detect smaller effects or associations. Increasing the sample size could enhance the robustness and reliability of the findings. The duration of our study, despite spanning several weeks, may have been insufficient to capture long-term changes or fully assess the effects of the interventions. A longer follow-up period could provide a more comprehensive understanding of participants’ responses over time. Additionally, it is important to note that most of our participants were females, which could limit the generalizability of the results to a broader population. Future studies will aim for more diverse participant demographics to ensure greater representation. Despite these limitations, this study provides valuable insights into the potential effects of the intervention.

## 5. Conclusions

Our study highlights new knowledge on the effects of virtual reality-based training on aging individuals’ balance ability and balance confidence. These findings have significant implications for the development of targeted interventions aimed at promoting healthy aging and reducing the risk of falls among older adults. By shedding light on the potential benefits of VR-based training, our research contributes to advancing the field of balance rehabilitation and underscores the importance of innovative approaches in improving the well-being of aging populations. Moving forward, maintaining or improving balance confidence and cognitive functioning alongside physical interventions remains an essential consideration in promoting overall well-being and healthy aging among older adult populations.

## Figures and Tables

**Table 1.** Overview of participant demographics (gray text indicates withdrawal from the study).

				Medical Conditions			Physical Abilities					Visual Abilities	
Subject	Age	Gender	Ethnicity	Traumatic Brain Injury	Dizziness/Vertigo	Other	Previous Falls	Meds Cause Dizziness/Loss of Balance	Exercise	Physical Limitations	Perceived Fitness Level (10 = Most, 1 = Least)	Glasses	Vision Loss
VR1	73	M	Caucasian	No	No	LTKR, lumbar spinal fusion	No	Yes	Yes (bike, swim, weights, stretch)	No	10	Yes	No
VR2	82	F	Caucasian	No	No	No	No	Y (Lunesta)	Yes (4×/wk, walk, yoga, weights)	No	10	No	No
VR3	78	F	Caucasian	No	No	Arthritis (hips/back)	No	No	No response	No response	No response	No response	No response
VR4	76	F	Caucasian	No	No	No	No	No	Yes (walk, weights, stretch)	No	6	Yes	No
VH5	77	F	Caucasian	No	No	No	No	No	Yes (walking)	Left shoulder issues	7	Yes	No
VR6	72	F	Black/African American	No	No	No	Yes (trip and fall of step)	No	Yes (bike 6×/wk, strength and stretch 2×)	No	6	Yes	No
VR7	NA	F	Caucasian	NA	NA	NA	NA	NA	NA	NA	NA	NA	NA
VR8	78	F	Caucasian	No	No	Mid-low back if sitting too long	No	No	Yes (walk, 10k steps/day, sometimes bike)	No	6	Yes	No
VR9	71	M	Caucasian	No	No	No	No	Yes (metformin, metoprolol)	Yes (6–7 days/wk of walking)	No	7	No	No
C1	78	F	Caucasian	No	No	No	No	No	Yes (walk several x/wk)	No	6	Yes	No
C2	79	F	Caucasian	No	No	Osteoporosis	Yes (once 2 mo ago)	No	Yes (2×/wk)	NA	6	Yes (post cataract surgery)	No
C3	82	F	Caucasian	No	No	No	Yes (on ice 3 yrs ago)	No	Yes (bike, walk, weights)	No	9	Yes (post cataract surgery)	No
C4	80	F	Caucasian	No	No	No	Yes (trip and fall while walking)	No	Yes (walk, weights, bike)	No	10	No (cataract surgery 5 yrs ago	No
C5	78	F	Caucasian	No	No	No	No	No	Yes (walking, bike)	No	6	Yes	No
C6	62	F	Black/African American	No	Yes	Osteoarthritis	No	No	Yes (5+ days, pool/other)	No	7	Yes	No
C7	73	F	Black/African American	No	No	No	No	No	Yes (5×/wk)	NA	6	NA	No
C8	69	F	Caucasian	No	No	No	No	No	Yes (4–5 days/wk, boxing, swimming, gym workout)	No	9	Yes	No
W	78	F	Moroccan	NA	No	No	No	No	Yes (walking)	NA	NA	Yes	No
W	72	F	Caucasian	No	No	No	Yes (trip and fall off step)	No	Yes (bike 6×/wk, strength and stretch 2×/wk)	No	6	Yes	No
W	NA	F	Black/African American	NA	NA	NA	NA	NA	NA	NA	NA	NA	NA
W	83	M	Caucasian	No	No	NA	Yes (1 mo ago, trip and broke nose)	No	No (last time 2 yrs ago)	No	4	Yes	No

## Data Availability

The data presented in this study are available on request from the corresponding author.
